# INVESTIGATING DYNAMICS OF AGE-ASSOCIATED TRANSCRIPTIONAL NETWORKS WITH INTERVENTIONS TARGETING AGING

**DOI:** 10.1093/geroni/igz038.2066

**Published:** 2019-11-08

**Authors:** Ameya S Kulkarni, Jessica C Mar, Nir Barzilai

**Affiliations:** 1 Albert Einstein College of Medicine, Bronx, New York, United States; 2 Australian Institute for Bioengineering and Nanotechnology, University of Queensland, Brisbane, Brisbane, Queensland, Australia

## Abstract

Biological aging is characterized by a progressive decline in physiological function from molecular to organismal levels, manifesting through adaptive transcriptional networks. We present an overview of the complex regulation of transcriptional networks in species- and tissue-specific aging. We aimed to: 1) capture the age-associated changes in gene-gene connectivity, and 2) evaluate the effect of two interventions targeting biological aging (metformin, acarbose) on the regulation of gene networks. Aim 1) Using RNA-Seq we modeled co-expression networks and identified differentially co-expressed gene-pairs between young, middle-aged and older-aged groups. Aim 2) Using short-term clinical studies in older humans (metformin: MILES-trial; acarbose: SAIL-trial), and complementary mouse studies, we revealed the genes and novel pathways underlying the drugs’ effects on biological aging in muscle and adipose. Importantly, these interventions shifted transcripts to a more youthful expression. Overall, we provide evidence of age-associated gene-network topology changes and identify upstream transcriptional factors affected by age-targeting drugs.

